# Rhizosphere and fruit microbiome variation across *Coffea canephora* farms representing conventional, transitional, and organic management

**DOI:** 10.3389/fmicb.2026.1899736

**Published:** 2026-09-22

**Authors:** Cham Thi-Mai Le, Fuki Fujiwara, Tai Thanh Nguyen, Hoa Anh Dinh, Phan Thi Tran, Trang Thuy Tran, Nguyet Thi-Anh Nguyen, Hiroshi Shiba, Mitsuru Tsubo, Kazuo N. Watanabe, Yasunori Ichihashi

**Affiliations:** 1 Division of Microbial Technology, Biotechnology Center of Ho Chi Minh City, Ho Chi Minh City, Vietnam; 2 RIKEN Center for Sustainable Resource Science (CSRS), Tsukuba, Japan; 3 Tsukuba Plant Innovation Research Center and Institute of Life and Environmental Sciences, University of Tsukuba, Tsukuba, Japan; 4 Arid Land Research Center, Tottori University, Tottori, Japan

**Keywords:** *coffea canephora*, fruit microbiome, organic transition, rhizosphere microbiome, Robusta coffee, sustainable agriculture

## Abstract

Robusta coffee (*Coffea canephora* Pierre ex A. Froehner) is a major global cash crop, and an increasing number of coffee farms are transitioning from conventional to organic management. To investigate how coffee-associated microbiomes vary across different management histories, bacterial communities from the rhizosphere and fruits of three farms representing conventional, transitional organic, and organic management were profiled using 16S rRNA gene amplicon sequencing, differential abundance analysis, and functional prediction. Differential abundance analysis of rhizosphere and fruit bacterial communities identified the largest number of differentially abundant taxa between the conventional and organic farms, fewer between the transitional organic and organic farms, and relatively few between the conventional and transitional organic farms. In the rhizosphere, these differences included taxa related to nitrogen cycling and contrasting soil resource conditions. Approximately 30% of bacterial taxa were shared between rhizosphere and fruit microbiomes, including lactic acid bacteria (*Lactobacillus plantarum*, *Leuconostoc mesenteroides*) and acetic acid bacteria (*Kozakia baliensis*, *Gluconobacter*) that are known to influence coffee fermentation. Shared genera accounted for more than 85% of the bacterial relative abundance detected in fruits, indicating a close correspondence between rhizosphere and fruit-associated bacterial communities. Although each management category was represented by only one farm, preventing the separation of management effects from farm-specific environmental and historical factors, these findings provide exploratory insights into microbiome variation among coffee farms with contrasting management histories and identify candidate microbial patterns for further validation in independently replicated studies.

## Introduction

Coffee (*Coffea* spp.) ranks among the most economically significant cash crops worldwide, sustaining livelihoods for millions in producing regions and driving substantial trade revenues (International Coffee Organization (ICO), 2024). Vietnam, as the world’s second-largest coffee producer, has a production profile dominated by *Coffea canephora* (Robusta coffee). Approximately 97% of the total coffee-growing area in Vietnam is dedicated to robusta, primarily cultivated in the Central Highlands, where favorable agroecological conditions and the crop’s inherent resistance to coffee leaf rust support large-scale production. In contrast, *Coffea arabica* (Arabica coffee), valued for its superior cup quality, is cultivated in limited quantities, grown primarily at higher altitudes in the Northwest regions and a specific area in the Central Highlands (Le et al., 2022; ICC-124-9, 2019). Vietnam’s average coffee yield is comparatively high, reaching 2.3 tons per hectare on average and up to 3.5 tons per hectare in optimal conditions, highlighting its efficiency and significance in the global coffee supply chain (ICC-124-9, 2019). With the rising demand for high-quality coffee in specialty markets, greater attention has been directed toward agronomic practices such as soil fertility and nutrient management. In recent years, an increasing number of coffee farms have transitioned from conventional high-input systems to organic management to align with consumer preferences and sustainable production goals.

The rhizosphere and root-associated microbiomes serve as a vital interface for coffee plants, governing nutrient cycling, stress tolerance, and pathogen defense (Berendsen et al., 2012; Velmourougane, 2016; Li et al., 2021; Pantigoso et al., 2022). Empirical studies indicate that fertilization regimes and long-term soil management significantly influence rhizosphere microbial diversity and structure (Veloso et al., 2020; Andrade et al., 2023). Marked differences in prokaryotic communities were observed among coffee plantations managed under conventional, organic, and transitional systems (Caldwell et al., 2015; Jurburg et al., 2020). Other factors such as genotype (de Sousa et al., 2022) and tree age (de Sousa et al., 2023) further shape rhizosphere microbiomes, underlining the dynamic and multifactorial nature of microbial community assembly. In addition to modulating nutrient dynamics, root microbiomes also bolster plant resilience under both abiotic stressors such as drought, salinity, low pH, and heavy metal exposure, and biotic pressures from pests and pathogens (Boubekri et al., 2022; Narsing Rao et al., 2022). Actinobacteria, a critical bacterial lineage in this context, enhance stress tolerance through multiple pathways, including phytohormone production, ACC-deaminase activity, siderophore release, and activation of host antioxidant systems (Palaniyandi et al., 2013).

In addition to the rhizosphere and root-associated microbiomes, the fruit microbiome plays a pivotal role in shaping coffee quality throughout postharvest processing (Elhalis et al., 2023). Several studies have demonstrated that fruit-associated microbiota strongly influence coffee fermentation and, consequently, flavor development (De Bruyn et al., 2017; Haile and Kang, 2019; Todhanakasem et al., 2024). During fermentation, regardless of whether starter cultures are added, the indigenous fruit microbiota significantly contribute to the biochemical transformations that define the sensory attributes of coffee (Avallone et al., 2001; Elhalis et al., 2021; Cruz-O'Byrne et al., 2021; Elhalis et al., 2023; Shen et al., 2025). The functional roles of fruit-associated microorganisms can be broadly divided into two aspects: degradation of mucilage polysaccharides through pectinolytic and cellulolytic enzymatic activities, facilitating bean processing; and generation of volatile and aroma-active metabolites that directly shape the final sensory profile of coffee (Elhalis et al., 2023; Shen et al., 2025). Notably, the fruit microbiome undergoes strong temporal fluctuations during fermentation, reflecting a dynamic succession of yeasts, lactic acid bacteria (LAB), and acetic acid bacteria (AAB) that drive distinct metabolic pathways (Elhalis et al., 2023; Shen et al., 2025).

While the rhizosphere and fruit microbiomes occupy distinct ecological compartments, both may be associated with plant performance and coffee quality. Understanding whether these compartments harbor overlapping bacterial communities may therefore provide insight into the organization of the coffee-associated microbiome. However, the extent of microbial overlap between belowground and aboveground compartments remains largely unexplored, especially in Robusta coffee cultivated in Vietnam. To address this gap, we performed 16S rRNA gene amplicon sequencing to characterize bacterial communities in coffee cultivated at three farms representing conventional, transitional organic, and organic management histories, with the aim of identifying compartment-specific microbial signatures and assessing the degree of overlap between rhizosphere- and fruit-associated microbiomes. This approach enables the identification of compartment-specific microbiome signatures and predicted functional potentials across the three farms. In addition, assessing microbial overlap between rhizosphere and fruit communities may provide insights into potential ecological links across coffee plant compartments.

## Materials and methods

### Study site and sampling

Samples were collected from coffee farms of Vietnam Coffee Academy Joint Stock Company (VCA), Vietnam with their geographic coordinates indicated in Figure 1 and Supplementary Table 1. All sites cultivated *Coffea canephora* var. TR4. Three distinct farms were screened, each characterized by specific cultivation practices and altitudes.

**Figure 1:**
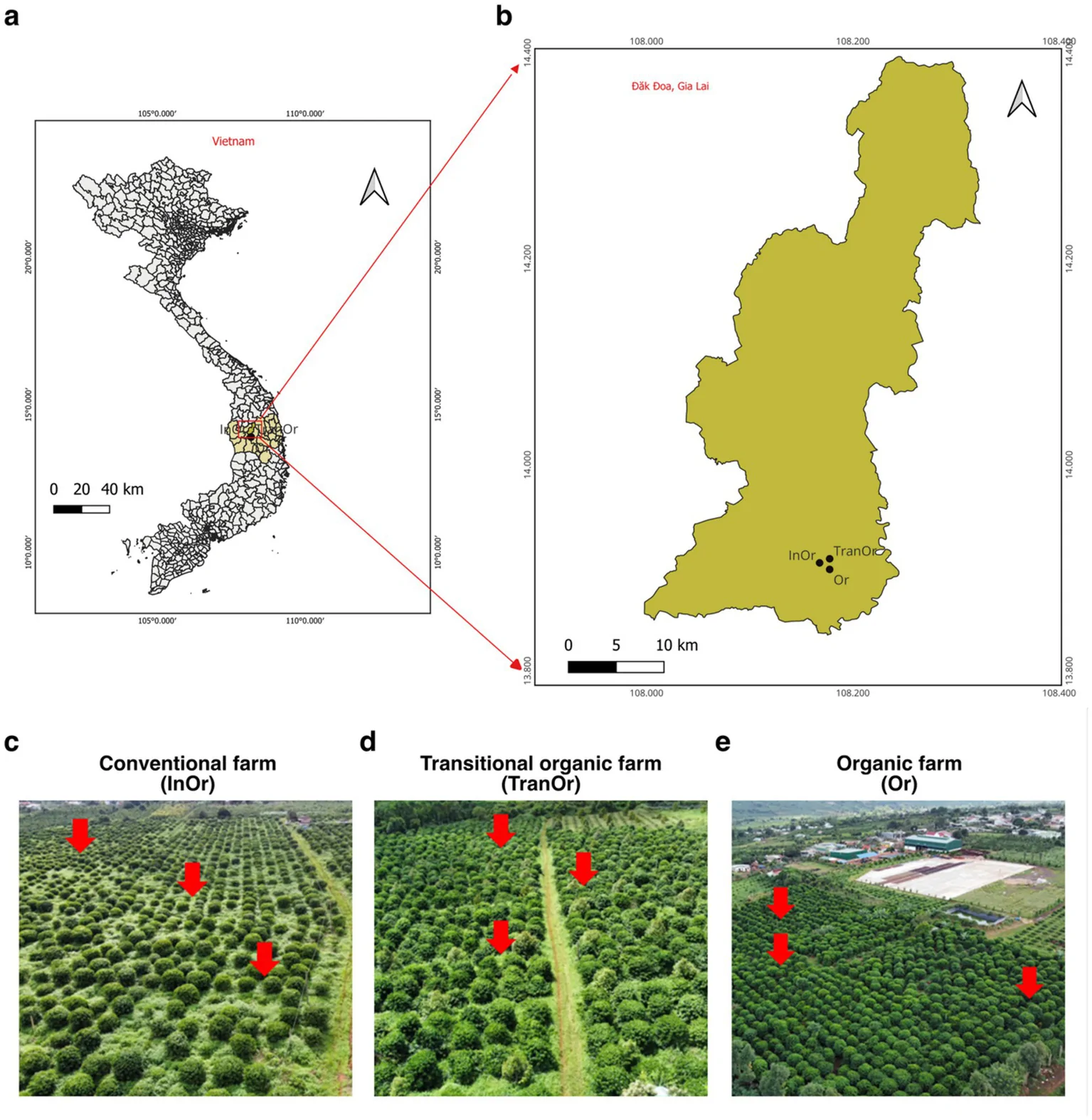
Sampling sites of Coffea canephora in Đăk Đoa, Gia Lai, Vietnam. **(a)** Location of Đăk Đoa District in Vietnam. **(b)** Locations of the three coffee plantations in Đăk Đoa District: Conventional farm (InOr), transitional organic farm (TranOr), and organic farm (Or). **(c–e)** Aerial views of the conventional, transitional organic, and organic farms, respectively. Red arrows indicate the locations where samples were collected within each plantation. Maps were generated using QGIS version 3.40.9.

Farm 1 (InOr; conventional farm) is situated at 725 m altitude and has been under conventional management for 10 years. Throughout this period, the fertilization regime consisted of annual inorganic applications supplemented by opportunistic dolomite and organic inputs. Pesticides were administered on an as-needed basis, triggered by the observed presence of pests or diseases (Supplementary Table 1). Farm 2 (TranOr; transitional organic farm) is located at 740 m altitude and has been cultivated for 12 years under primarily inorganic fertilization practices, with pesticides applied contingent upon the occurrence of pests or diseases in the plantation. Two years prior to sampling (2022), durian trees were introduced as an intercrop within the coffee plantation. Subsequently, in 2023, the farm transitioned to organic management, with the application of mineral–organic fertilizers (Hapharchem Agricare Co., Ltd., Vietnam) and a single annual input of composted cow manure in September. Dolomite is typically applied to the durian trees in the farm at the beginning of the rainy season (May–June) to improve soil pH (Supplementary Table 1). Farm 3 (Or; organic farm) is a monoculture coffee field positioned at approximately 750 m above sea level, planted for 12 years before sampling. During the first 9 years, the plantation was managed under conventional practices, characterized by predominantly inorganic fertilization similar to other coffee farms in Vietnam. In 2020, the farm transitioned to organic management, adopting mineral–organic fertilizers (Hapharchem Agricare Co., Ltd., Vietnam) and a single annual application of composted cow manure (the same fertilizers as Farm 2). No synthetic pesticides have been used for pest or disease control since 2020. With this practice, the plantation was converted to organic farming and received USDA organic certification in 2024.

All sampled coffee trees were mature plants. Trees in the conventional farm were approximately 10 years old, whereas those in the transitional organic and organic farms were approximately 12 years old (Supplementary Table 1). All samples were collected during the same coffee harvest period using a standardized sampling protocol to minimize variation associated with sampling date and plant developmental stage and to facilitate direct comparisons among farms. Samples from all three farms were collected and initially processed within the same day to minimize potential variation associated with sampling time and sample handling. At each plantation, three distinct sampling sites were established as described by Veloso et al. (2020), illustrated in Supplementary Table 1 and Figure 1. From each site, three proximate trees were selected for sampling. For rhizosphere sampling, soil was excavated from three positions beneath the coffee canopy where fine roots were easily found. Soil and fine roots from each tree were collected using sterilized shovels and subsequently pooled across the three trees into a single sterile nylon bag to form one sample. Fine roots were then manually separated, cut into small fragments using sterilized scissors, and preserved in DESS solution for downstream analysis (Barros et al., 2024).

Soil pH was determined in a 1:2.5 (w/v) soil-to-distilled water suspension. Briefly, 10 g of air-dried soil was mixed with 25 mL of distilled water, shaken for 60 min, allowed to equilibrate for 60 min, and stirred immediately before measurement using a calibrated pH meter (Mettler Toledo, Switzerland). For soil nutrient analysis, composite soil samples were prepared by pooling soil from each sampling site within a farm and homogenizing the mixture, then submitted to Eurofins ETM (Ho Chi Minh City, Vietnam) for analysis. Soil physicochemical properties were determined by an accredited analytical laboratory using standardized analytical methods. Electrical conductivity (EC) was measured in a soil–water suspension, while total organic carbon (TOC) and total nitrogen (TN) were determined according to standardized chemical analysis procedures. Total phosphorus (P), available phosphorus (Bray I), exchangeable potassium (K), exchangeable calcium (Ca), magnesium (Mg), sodium (Na), and potassium (K) were quantified using established soil analytical methods and instrumental analyses appropriate for each parameter. The analytical procedures followed Vietnamese National Standards (TCVN), ISO standards, U. S. EPA methods, FAO recommendations, and the Soil Chemical Methods–Australasia protocols, as appropriate for each measurement (Supplementary Table 2).

For coffee cherry sampling, approximately 10 fruits per tree were harvested and pooled across three trees at each sampling site to generate one composite sample, then placed in sterile polyethylene bags. Coffee cherries were processed without surface sterilization because the objective of this study was to characterize the naturally occurring fruit-associated bacterial community under field conditions rather than to specifically target endophytic microorganisms. Each fruit was dissected with five incisions using a sterilized scalpel and preserved in DESS solution for downstream analyses. Sterile gloves were replaced between each sampling site to prevent cross-contamination. Rhizosphere and fruit samples were collected independently by different personnel during the sampling.

Samples were labeled as InOrx for conventional farm, TranOrx for transitional organic farm, and Orx for organic farm, where “x” denotes the specific sampling site within each field. All samples designated for 16S rRNA gene amplicon analysis were preserved in DESS solution and maintained at ambient temperature until subsequent laboratory processing.

### DNA extraction

Each biological sample was preserved in 50 mL Falcon tubes (JetBiofil®, Guangzhou Jet Bio-Filtration Co., Ltd., China) with DESS solution and vortexed for 5 min to ensure sample homogeneity. Visible plant debris was manually removed prior to centrifugation at 4,500 rpm for 15 min at 4 °C. After centrifugation, the supernatant was discarded, and the residual pellet was resuspended in 500 μL of sterile Milli-Q water. The tubes were immediately stored at −80 °C prior to DNA extraction, thereby minimizing the duration of exposure to hypotonic conditions. Mechanical disruption of cells was performed using glass beads at 50 Hz for 5 min with a TissueLyser LT (QIAGEN, Hilden, Germany). Total DNA was subsequently extracted using the Extrap Soil DNA Kit Plus version 2 (Nippon Steel Eco-Tech Corporation, Japan), following the manufacturer’s standard protocol. The procedure encompassed cell lysis through bead beating in lysis buffer, protein precipitation, and magnetic bead-based DNA purification. DNA concentration was measured using fluorescence-based quantification with a Qubit fluorometer (Thermo Fisher Scientific, USA), while purity was evaluated spectrophotometrically with a NanoDrop spectrophotometer (Thermo Fisher Scientific, USA). Samples were considered suitable for library preparation when the concentration was ≥ 0.20 ng/μL, total DNA amount ≥ 15.00 ng, and OD260/OD280 ratio ≥ 1.50.

### Library preparation

DNA samples that met quality control criteria were submitted to KTest Science Co., Ltd. (Vietnam) for sequencing. Bacterial community profiling was performed using the IDT xGen™ 16S Amplicon Panel v2 (Integrated DNA Technologies, Coralville, IA, USA), which uses multiplexed tiled primer pairs targeting the V1–V9 variable regions of the 16S rRNA gene. Libraries were sequenced using paired-end 2 × 150 bp chemistry according to the manufacturer’s protocol. Library concentration was quantified by qPCR using the KAPA Library Quantification Kit for Illumina® Platforms (Cat. No. KR0405), and only libraries with a concentration ≥ 0.30 nM were selected for sequencing. High-throughput sequencing was performed using paired-end 150 bp reads (150PE) on the DNBSeq-G99 platform (MGI Tech Co., Ltd), with an optimal read depth of 100,000 reads per sample for downstream 16S rRNA gene amplicon analysis.

### Bioinformatic and statistical analysis

Raw sequence data underwent initial quality control and adapter trimming using Trimmomatic version 0.39 and Cutadapt version 2.10. High-quality reads were denoised and grouped into amplicon sequence variants (ASVs) using the q2-DADA2 plugin (Callahan et al., 2016). Representative ASVs were classified using the SILVA database version 138 SSURef Nr99 (Quast et al., 2013) via the q2-feature-classifier plugin with the classify-consensus-blast method. The resulting feature tables and taxonomic assignments were used for downstream ecological analyses. Predicted functional profiles were inferred from 16S rRNA gene amplicon data using PICRUSt2 version 2.3.0-b (Douglas et al., 2020) with the MetaCyc database. This approach predicts gene content and metabolic pathway profiles based on 16S rRNA gene sequences. All analyses were performed using default parameters unless otherwise specified.

Alpha diversity metrics were calculated to evaluate within-sample diversity, including Observed features (Gotelli and Colwell, 2001), Chao1 richness estimator (Chao, 1984), Shannon diversity index (Shannon, 1948), and Pielou’s evenness (Pielou, 1966). The Chao1 index is sensitive to rare taxa, whereas Pielou’s evenness quantifies the homogeneity of taxon abundance distributions (range: 0–1). Observed Features reflected the number of distinct ASVs per sample, and the Shannon index provided an integrated measure of both richness and abundance distribution. One-way ANOVA and post-hoc Tukey HSD test were performed to test the statistical difference among the three farms.

Beta diversity was assessed to compare microbial community composition and its functional profile across samples using Bray–Curtis dissimilarity (Bray and Curtis, 1957), calculated with the vegan package version 2.6–10 in R. The Bray-Curtis index (0 = identical communities, 1 = completely dissimilar) accounts for both shared taxa and their relative abundances. Principal coordinates analysis (PCoA) (Gower, 1966) was applied for ordination, and statistical significance of group-level differences was tested using PERMANOVA (Anderson, 2001) with the vegan package.

Differential abundance analysis at the species level among the three farms was conducted using the ALDEx2 package version 1.32.0 in R version 4.1.2 (R Core Team, 2021). ALDEx2 accounts for the compositional nature of sequencing count data by applying a centered log-ratio (CLR) transformation and Monte Carlo sampling from the Dirichlet distribution (Fernandes et al., 2013). Because the number of replicates per farming practice was limited (*n* = 3), we followed the results of Fernandes et al. (2018) that effect size–based detections provide more reproducible results than *p*-value–based testing in small-sample cases. Accordingly, differentially abundant species were identified based on combined criteria of absolute effect size > 1, absolute difference > 1, and an effect-size confidence interval not crossing zero.

To compare the overlap of microbial taxa between rhizosphere and fruit microbiomes, Venn diagrams were generated for each farm (InOr, TranOr, and Or). At the species level, a taxon was considered present within a community (rhizosphere or fruit) if it was detected in at least one of the three replicate samples. Venn diagrams were then used to illustrate the numbers and proportions of unique and shared species between rhizosphere and fruit under each farming practice. To further evaluate whether the shared taxa represented a major component of the fruit microbiome in terms of relative abundance, fruit-associated genera were classified as “shared” if they were also detected in the rhizosphere, or as “fruit only” if they were detected exclusively in fruit samples. Mean relative abundances were calculated for each farming practice, and the top 30 most abundant shared genera were visualized individually.

### Coffee bean extraction and compound identification workflow

Fruit samples used for chemical compound analysis were collected simultaneously with the samples used for microbiome analysis to ensure consistency between metabolite and microbial datasets. At each sampling site, approximately 500 g of coffee fruits were collected from three trees (pooled), resulting in a total of ~1,500 g per farm (three sampling sites). The pooled fruits from each farm were processed separately and separated into beans and outer skins. Beans were dried at 60 °C to a moisture content of ~12–15%. Dried beans were ground to a fine powder, and subsamples were taken in triplicate from each farm for metabolite extraction. For each replicate, 3 g of powder was sonicated in 80% methanol containing 0.1% formic acid, followed by centrifugation, filtration, and two successive extractions. The combined supernatants were concentrated using a rotary evaporator (Buchi R-300, Switzerland) and reconstituted in methanol prior to 0.22 μm filtration. Extracts were subjected to UPLC-QToF-MS/MS analysis (Waters Corp., USA). Chromatographic separation was performed on a Waters Acquity UPLC HSS T3 column with a water/acetonitrile gradient containing 0.1% formic acid. Mass spectrometry was carried out in positive electrospray ionization (ESI^+^) mode across a scan range of 100–1,200 Da, with optimized ionization and fragmentation settings.

Data were processed with UNIFI NPAS software (Waters Corp., USA) for spectral alignment, peak deconvolution, and compound annotation against multiple libraries (Green Tea Library_Updated; University of Mississippi, University of Ottawa Natural Products Libraries; and an in-house library). Processed outputs were formatted for MetaboAnalyst^1^ (Chong et al., 2019) by R version 4.1.2 (R Core Team, 2021). Filtering included blank subtraction (sample/blank ≥ 10), RSD ≤ 20%, exclusion of the bottom 25% by IQR, and removal of features with mean intensity = 0. Data normalization applied cube root transformation and Pareto scaling (van den Berg et al., 2006). PLS-DA was conducted with VIP scores calculated to determine key discriminating compounds, where VIP > 1 indicates strong influence (Barker and Rayens, 2003; Chong and Jun, 2005; Westerhuis et al., 2010).

## Results

### Physicochemical characteristics and nutrient status of soil samples

Soil chemical properties varied among the farms (Table 1; Supplementary Table 3). Soil pH values for individual sampling points are presented in Supplementary Table 3, whereas the summarized soil physicochemical and nutrient characteristics of the three coffee farms are presented in Table 1. Soil pH ranged from 5.14 to 5.87 across the three farms. EC ranged from 0.189 to 0.292 mS/cm. Magnesium concentration was highest in TranOr (1,230 mg/kg), followed by InOr (1,010 mg/kg) and Or (619 mg/kg). Exchangeable K ranged from 0.34 to 0.79 cmolc/kg. TOC ranged from 3.69 to 4.80%, with the highest value in Or. Both total and available phosphorus were higher in Or than in InOr and TranOr. Sodium concentrations were below 100 mg/kg in all three farms.

**Table 1:** Soil physicochemical and nutrient characteristics of the three coffee farms.

No.	Parameters	Unit	Results		
			InOr	TranOr	Or
1	Average soil pH		5.23	5.87	5.14
2	Electrical conductivity (EC)	mS/cm	0.189	0.230	0.292
3	Total organic carbon (TOC)	%	3.89	3.69	4.80
4	Total nitrogen	mg/kg	3,060	2,640	3,510
5	Total phosphorus	mg/kg	474	880	2,200
6	Available P (Bray I)	mg/kg	18.2	63.6	112
7	Potassium (K)	mg/kg	253	279	407
8	Exchangeable potassium	cmol c/kg	0.34	0.44	0.79
9	Exchangeable Calcium	cmol c/kg	3.18	0.51	8.34
10	Sodium (Na)	mg/kg	Traces (<100)	Traces (<100)	Traces (<100)
11	Magnesium (Mg)	mg/kg	1,010	1,230	619

### Diversity of bacterial communities associated with the coffee rhizosphere and fruit

Sequencing generated 248,156–398,902 raw reads per sample. After quality filtering, 143,855–222,676 high-quality reads per sample were retained for subsequent DADA2 processing and downstream taxonomic and diversity analyses (Supplementary Table 4). Alpha diversity of rhizosphere microbiome was assessed using four complementary metrics: Chao1 richness, observed features, Shannon diversity, and Pielou’s evenness. InOr samples exhibited the highest richness, TranOr intermediate, and Or the lowest, a trend consistent across Shannon, observed features, and Chao1 metrics (Figure 2a; Supplementary Figure 1; Supplementary Table 5). Pielou’s index suggested slightly greater evenness in InOr samples, though less pronounced (Supplementary Figure 1). Although these differences were not statistically significant (*p* > 0.05), InOr samples showed a tendency toward higher richness and evenness, whereas Or samples tended to show lower diversity.

**Figure 2:**
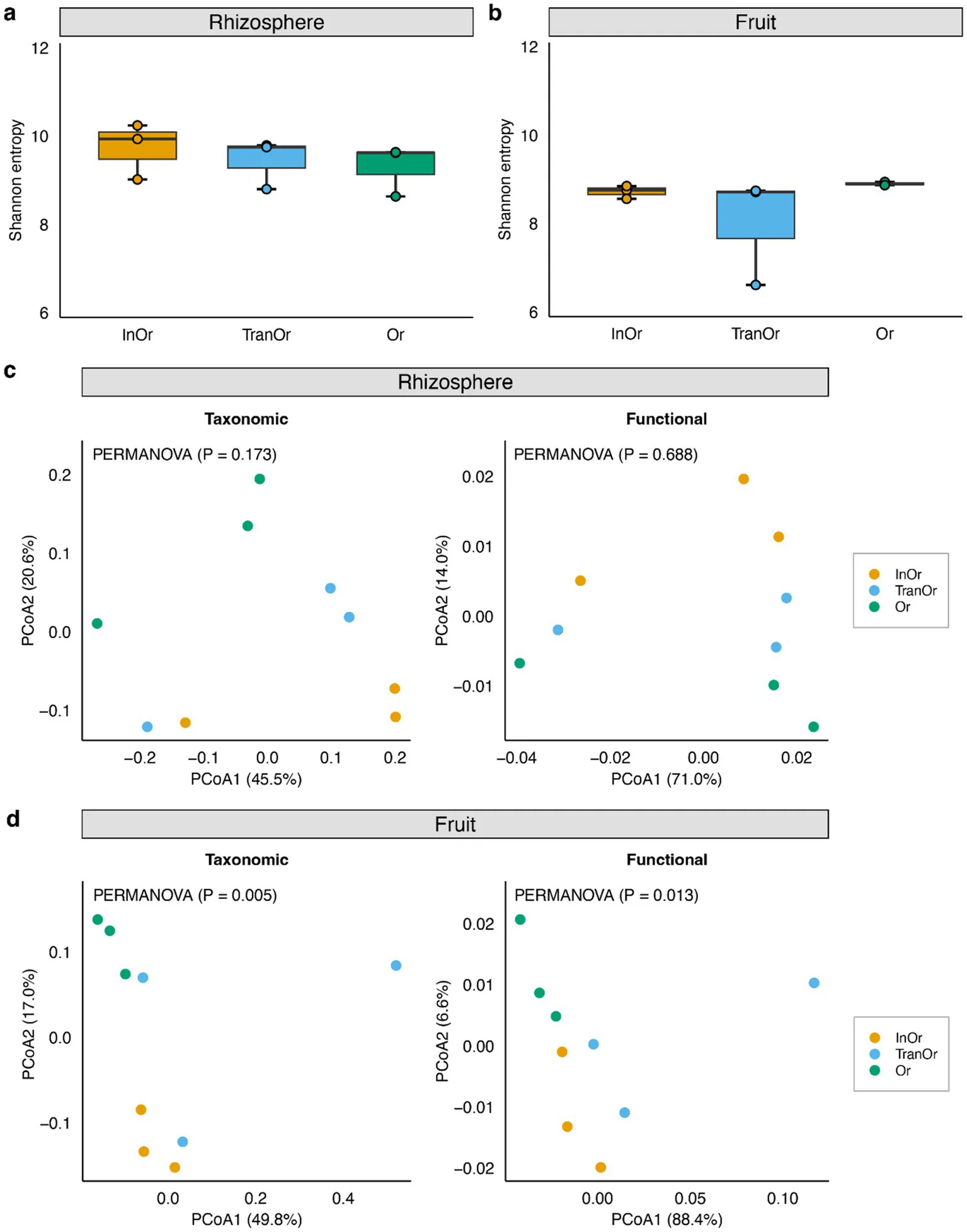
Diversity of bacterial communities associated with the coffee rhizosphere and fruit: **(a)** Shannon entropy of rhizosphere microbiomes; **(b)** Shannon entropy of fruit microbiomes; **(c)** Principal coordinate analysis (PCoA) plots based on beta diversity of taxonomic composition and predicted functional profiles of rhizosphere bacterial communities; **(d)** Principal coordinate analysis (PCoA) plots based on beta diversity of taxonomic composition and predicted functional profiles of fruit-associated bacterial communities across conventional, transitional organic, and organic farms. Sample size per group: *n* = 3. Statistical comparisons of alpha diversity among farms were performed using Tukey’s HSD test. Percentages on PCoA axes indicate the proportion of variation explained by each coordinate. Differences among farms were assessed using PERMANOVA.

On the other hand, alpha diversity of fruit microbiome revealed a descending trend in bacterial richness, with Or samples exhibiting the highest number of species, followed by InOr and TranOr, although these differences were not statistically significant (*p* > 0.05, Figure 2b; Supplementary Figure 1; Supplementary Table 6). While overall richness and diversity varied among the farms, evenness values remained relatively similar across all three groups (*p* > 0.05).

Comparison of alpha diversity across the rhizosphere and fruit-associated microbiome revealed significant variation between the two compartments (*p* < 0.05, Supplementary Figure 2). Rhizosphere supported a more taxonomically diverse microbial community compared to fruit, largely due to the enrichment of both rare and common bacterial taxa, while fruit was characterized by lower richness but greater uniformity in taxa distribution (Supplementary Figure 2). The results demonstrate that microbial diversity patterns are compartment-specific, with the rhizosphere consistently harboring a richer community than the fruit.

Beta diversity of the rhizosphere microbiome was assessed using Principal Coordinates Analysis (PCoA) based on Bray–Curtis distance metrics (Figure 2c). No significant differences in taxonomic composition were observed among the three farms (PERMANOVA, *p* = 0.173), as samples from InOr, TranOr, and Or were broadly dispersed and partially overlapped on the PCoA plot. Nevertheless, the Or group showed a slight tendency to separate along the PCoA2 axis, suggesting modest shifts in microbial community structure that were not strong enough to produce statistically significant differences. Similarly, functional profiles were not significantly different among the three farms (PERMANOVA, *p* = 0.688, Figure 2c). However, the three groups also exhibited a slight separation trend along the PCoA2 axis, indicating subtle functional shifts in the rhizosphere microbiome associated with different farms.

For fruit-associated bacterial communities, PCoA analysis revealed clear differences in both the taxonomic composition and functional potential of fruit-associated bacterial communities across the three farms (PERMANOVA, *p* < 0.05, Figure 2d). The most pronounced separation was observed between InOr and Or, whereas TranOr did not form a distinctly separated cluster. These results indicate significant differences in taxonomic composition and predicted functional profiles among the three sampled farms. In summary, while rhizosphere bacterial communities did not differ significantly between the three farms, fruit-associated communities displayed clear and significant differences.

### Changes in coffee rhizosphere and fruit microbiomes among the three farms

Beyond community-level diversity, we further investigated how individual bacterial taxa varied in abundance among the three sampled farms. At the phylum level, rhizosphere bacterial communities across all samples were consistently dominated by *Proteobacteria* (25–45%), followed by Acidobacteriota and Actinobacteriota (10–20% each). Bacteroidota was moderately abundant (5–15%), while other phyla such as Chloroflexi and Verrucomicrobiota appeared at lower levels (~5–10%). Minor phyla including Planctomycetota, Myxococcota, Patescibacteria, and Gemmatimonadota collectively contributed only a few percent (Figure 3a). Among identified genera, *Burkholderia-Caballeronia-Paraburkholderia* (3–7%) and *Acidibacter* (2–5%) were consistently detected. Other genera (Subgroup_2, *Sphingobium*, *Acidothermus*, *Rhodanobacter*, *Solibacter*, *Bradyrhizobium*, *Sphingomonas*, *Streptomyces*) appeared sporadically and at low abundance (< 5%) (Supplementary Figure 3; Supplementary Table 5).

**Figure 3:**
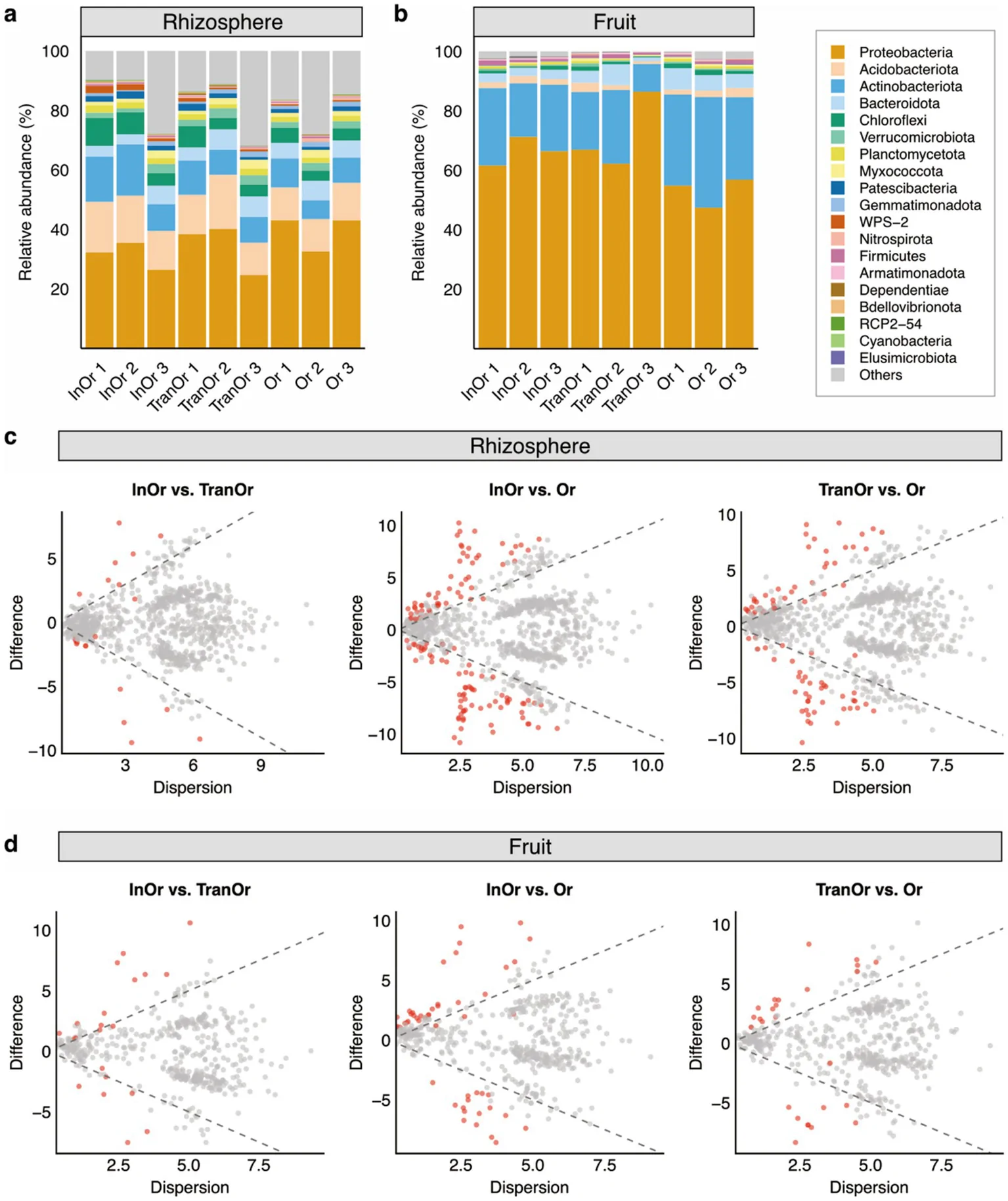
Relative abundance and difference in coffee rhizosphere and fruit bacterial communities among the three farms. **(a)** Relative abundance of the top 20 phyla in rhizosphere microbiomes; **(b)** Relative abundance of the top 20 phyla in fruit-associated microbiomes; **(c)** Differential abundance of rhizosphere microbiomes between each pair of the three farms; **(d)** Differential abundance of fruit-associated microbiomes between each pair of the three farms. In panels **a** and **b**, only the 20 most abundant taxa are shown individually, and all remaining taxa are grouped into the “Others” category for visualization. In panels **c** and **d**, positive difference values indicate higher abundance in the first farm listed, whereas negative values indicate higher abundance in the second farm listed. Red points denote taxa meeting all three criteria: Absolute effect size > 1, absolute difference > 1, and effect-size confidence interval not crossing zero. Grey points indicate taxa that did not meet all three criteria. Dashed lines represent *y* = ±*x*.

Differential abundance analysis using ALDEx2 revealed distinct microbial taxa in the rhizosphere among the three farms. Comparative analysis indicated pronounced differences between TranOr and Or, as well as between InOr and Or, whereas InOr and TranOr showed relatively few differentially abundant features (Figure 3c; Supplementary Table 7). At the species-level, ALDEx2 identified 22 differentially abundant taxa between InOr and TranOr, 80 between TranOr and Or, and 128 between InOr and Or (Figure 3c). At the genus level, representative taxa with higher abundance in Or than in InOr included *Nitrospira*, Nitrosomonadaceae MND1, *Gaiella*, and *Streptomyces*, whereas B12-WMSP1, *Acidothermus*, and *Crossiella* showed higher abundance in InOr (Supplementary Table 7).

PICRUSt2-based differential abundance analysis identified 18 predicted pathways differing between InOr and Or, two between InOr and TranOr, and five between TranOr and Or (Supplementary Table 8). In the InOr–Or comparison, 10 pathways showed higher predicted relative abundance in Or. Nine of these were associated with aromatic compound degradation, including protocatechuate degradation (P184-PWY), gallate degradation (GALLATE-DEGRADATION-I-PWY and GALLATE-DEGRADATION-II-PWY), methylgallate degradation (METHYLGALLATE-DEGRADATION-PWY), vanillin/vanillate degradation (PWY-7097, PWY-7098, and PWY-6338), benzoyl-CoA degradation (PWY-1361), and syringate degradation (PWY-6339). In contrast, eight pathways showed higher predicted relative abundance in InOr (Supplementary Table 8).

In contrast to the rhizosphere, Proteobacteria emerged as the predominant phylum in fruit-associated communities, consistently accounting for most sequences across samples (Figure 3b). Their relative abundance typically ranged from around 50% to over 70%, indicating a strong phylum-level dominance in this niche. The second most abundant phylum was Actinobacteriota, followed by Acidobacteriota, although their contributions varied considerably across different samples. Other phyla, including Bacteroidota, Chloroflexi, Verrucomicrobiota, and Firmicutes, were detected at lower relative abundances, generally below 10%. At the genus level, fruit samples were dominated by a narrower set of taxa, most notably *Methylobacterium-Methylorubrum*, *Enterobacter*, *Actinomycetospora,* 1,174–901-12, *Sphingomonas*, *Mycobacterium*, and *Spirosoma* (Supplementary Figure 3; Supplementary Table 6). TranOr3 showed a relatively high abundance of *Enterobacter* compared with the other fruit samples (Figure 3b).

Comparative analysis of fruit-associated bacterial communities using ALDEx2 identified relatively few differentially abundant taxa between InOr and TranOr, whereas more taxa differed between TranOr and Or and between InOr and Or. At the species-level feature resolution, ALDEx2 identified 21 differentially abundant features between InOr and TranOr, 26 between TranOr and Or, and 50 between InOr and Or (Figure 3d). At the genus level, representative taxa with higher abundance in InOr than in Or included *Serratia*, *Leifsonia*, *Burkholderia-Caballeronia-Paraburkholderia*, *Friedmanniella*, *Deinococcus*, *Luteibacter*, *Enterobacter*, and *Rhodanobacter*, whereas *Cryptosporangium*, *Haliangium*, *Sandaracinobacter*, *Dactylosporangium*, WPS-2, and *Kineosporia* showed higher abundance in Or (Supplementary Table 9).

To assess potential differences in metabolite compositions in coffee beans across the farms, metabolite profiling was conducted using UPLC-QToF-MS/MS, followed by compound annotation (Supplementary Table 10) and multivariate analysis via PLS-DA (Supplementary Figure 4) with VIP scoring. Because the three analytical replicates for each farm were derived from a single pooled biological sample, these results should be regarded as exploratory and should not be interpreted as statistically validated farm-level differences (Supplementary Figures 5, 6; Supplementary Table 11). The pooled InOr sample showed relatively higher abundances of several secondary metabolites, including alkaloids (caffeine, trigonelline), phenolic acids (methyl chlorogenate, 1,5-O-dicaffeoylquinic acid), coumarins (umbelliferone, dihydrocoumarin), and cyanogenic glycosides (lotaustralin), whereas relatively lower abundances of these compounds were observed in the pooled Or sample.

### Overlap between coffee rhizosphere and fruit-associated microbiomes

To better understand the relationships between belowground and aboveground microbiomes, we constructed Venn diagrams to compare and identify overlapping bacterial taxa between rhizosphere and fruit communities within each of the three farms. The results revealed 250 shared taxa (approximately 29%) in InOr, 241 shared taxa (28%) in TranOr, and 269 shared taxa (30%) in Or, suggesting that the proportion of overlapping taxa consistently hovers around 30% (Figure 4a). These shared bacterial groups included lactic acid bacteria (LAB), such as *Lactobacillus* spp. and *Leuconostoc* spp.; acetic acid bacteria (AAB), including *Gluconobacter*, *Kozakia baliensis*, and *Acidiphilium*; as well as methylotrophic and C1-metabolizing bacteria, such as *Methylorubrum* spp., members of the Beijerinckiaceae, and *Novosphingobium*. In addition, filamentous *Actinomycetes* (*Streptomyces*, *Actinoplanes*) and a variety of plant growth–promoting and nitrogen-fixing bacteria (*Rhizobium* sp., *Paraburkholderia*, *Bradyrhizobium*, *Pantoea*, *Bacillus*, *Pseudomonas*) were identified (Supplementary Table 12).

**Figure 4:**
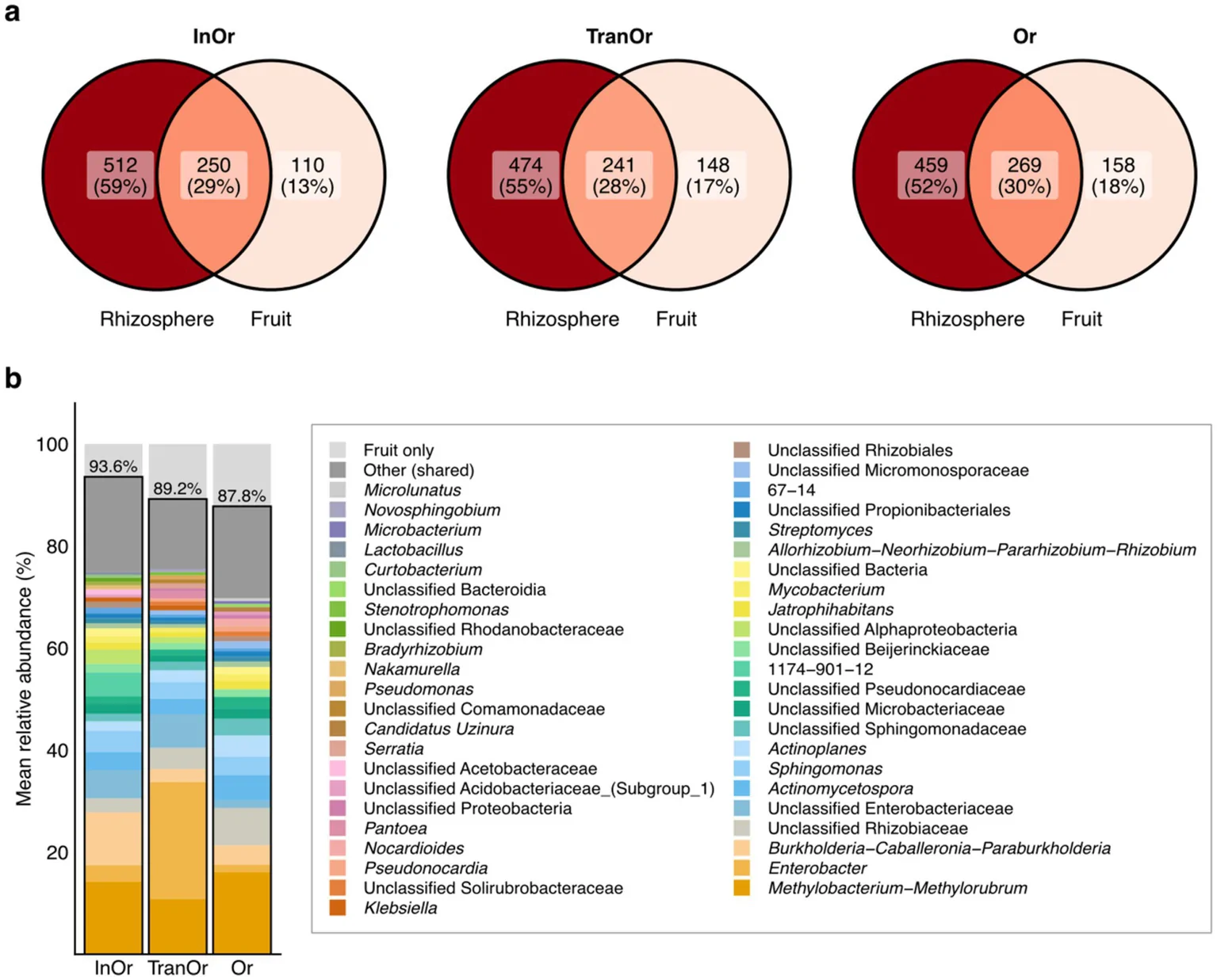
Overlap of rhizosphere and fruit-associated bacterial communities across three farms. **(a)** Venn diagrams showing the numbers and proportions of taxa in rhizosphere and fruit bacterial communities. **(b)** Relative abundance of bacterial genera in the coffee fruit bacterial community classified by occurrence in rhizosphere soil. Values in the Venn diagrams indicate the numbers of unique or shared taxa, with percentages calculated relative to the total taxa detected in each system. The orange section represents the proportion of bacterial taxa shared between rhizosphere and fruit microbiomes, whereas the red and light orange sections indicate taxa uniquely detected in the rhizosphere and fruit compartments, respectively. Stacked bar charts show the mean relative abundance (%) of bacterial genera detected in the coffee fruit microbiome across the three farms: Conventional (InOr), Transitional Organic (TranOr), and Organic (Or). Genera are classified as “shared” (shared between rhizosphere soil and fruit microbiome) or “fruit only” (exclusive to the fruit microbiome). Within the shared genera, the top 30 most abundant genera are shown individually, with the remaining shared genera grouped as “Other (shared).” The black outline and percentage value above it indicate the total relative abundance of shared genera for each farm. Mean values were calculated from three samples per farm.

To assess the extent to which bacterial genera detected in fruit samples were also detected in the rhizosphere, we quantified the relative abundance of shared genera at the genus level (Figure 4b). Across the three farms, shared genera accounted for more than 85% of the total bacterial relative abundance detected in the fruit microbiome, indicating that a substantial proportion of the fruit-associated bacterial community was composed of genera also present in the rhizosphere. Among the farms, InOr exhibited the highest proportion of shared genera (93.6%), followed by TranOr (89.2%), whereas Or showed the lowest proportion (87.8%). Among these shared genera, *Methylobacterium*-*Methylorubrum*, *Enterobacter*, and *Burkholderia*-*Caballeronia*-*Paraburkholderia* were particularly dominant across the fruit microbiome. Notably, *Lactobacillus*, a bacterial genus involved in coffee fermentation and flavor development, was also detected among the genera shared between rhizosphere and fruit samples. These results indicate that rhizosphere-shared genera represented a quantitatively major fraction of the fruit microbiome. This pattern suggests that the extent of rhizosphere–fruit microbiome association may differ among the examined farms.

## Discussion

### Rhizosphere bacterial community variation among farms with contrasting management histories

Our findings revealed differences in bacterial community composition among the three coffee farms with contrasting management histories. This observation is consistent with previous studies highlighting the strong influence of fertilizer regimes on coffee root microbiomes (Veloso et al., 2020; Andrade et al., 2023). Soil chemical analyses in this study indicated that the Or farm showed higher measured concentrations of N, P, K, and TOC than the InOr and TranOr farms (Table 1), suggesting that differences in soil nutrient conditions may contribute to shifts in bacterial community composition. Differential abundance analysis using ALDEx2 identified different numbers of differentially abundant taxa at the species level among the pairwise farm comparisons: 22 taxa between InOr and TranOr, 80 between TranOr and Or, and 128 between InOr and Or (Figure 3c; Supplementary Table 7). The fruit-associated microbiome also showed the largest number of differentially abundant features between InOr and Or, indicating that the pronounced contrast between these two farms was not restricted to the rhizosphere (Figure 3d; Supplementary Table 9). The relatively small differences observed between the conventional and transitional organic farms may partly reflect the persistence of legacy effects from previous long-term inorganic fertilization, consistent with evidence that soil microbial communities can change progressively during conversion to organic management (van Rijssel et al., 2022). Accordingly, the organic farm included in this study may represent a later stage of transition rather than a fully equilibrated endpoint of soil ecological recovery. These patterns are consistent with the possibility that microbial community restructuring during organic conversion may occur gradually rather than as an immediate response to management change. However, because each management category was represented by a single farm, these differences cannot be interpreted as evidence of a temporal trajectory of organic conversion. Given the limited sample size, the absence of significant differences in alpha diversity may partly reflect limited statistical power and should therefore not be interpreted as evidence of ecological equivalence among the sampled farms. In addition, although all sampled plants were mature coffee trees, minor differences in plant age among farms may also have contributed to the observed microbiome variation and therefore cannot be completely excluded.

Focusing on the comparison between InOr and Or, the contrasting bacterial taxa may reflect differences in soil resource conditions between the two farms. In Or, *Nitrospira* and Nitrosomonadaceae MND1 were more abundant (Supplementary Table 7). Members of *Nitrospira* include nitrite-oxidizing and complete ammonia-oxidizing bacteria (Daims et al., 2015; Li et al., 2020), and MND1 is a member of Nitrosomonadaceae, a family containing many ammonia-oxidizing bacteria (Tang et al., 2023). Together with the higher measured soil N concentration in Or (Table 1), these patterns may reflect differences in nitrogen-cycling communities. In addition, Or showed higher abundances of *Gaiella* and *Streptomyces*, whose members include taxa capable of utilizing or decomposing diverse organic substrates (Albuquerque et al., 2011; Severino et al., 2019; Crawford, 1978). In contrast, B12-WMSP1, *Acidothermus*, and *Crossiella* were more abundant in InOr. These taxa or their related lineages have been reported from diverse terrestrial environments, including soils with relatively low nutrient availability (Yabe et al., 2017; Zheng et al., 2019; Bossolani et al., 2021). Given the observed differences in measured N, P, K, and TOC concentrations between the farms, their higher abundance in InOr may therefore be consistent with the relatively lower soil resource levels observed at this farm. Although several of the differentially abundant taxa occurred at low relative abundance, low-abundance soil bacteria may nevertheless contribute to ecosystem functioning through diverse metabolic capacities (Kurm et al., 2017), while noncore bacterial taxa have been associated with soil nutrient cycling and microbial network stability in agricultural ecosystems (Ye et al., 2022). Collectively, these patterns suggest that differences in the measured soil chemical properties between farms were accompanied by contrasting bacterial taxa with distinct ecological characteristics, but their proposed functional roles remain hypotheses requiring direct validation.

Predicted functional profiles provided a complementary view of the taxonomic differences described above. Consistent with the differential abundance patterns of bacterial taxa, the largest number of differentially abundant predicted pathways was detected between InOr and Or, whereas only two were detected between InOr and TranOr and five between TranOr and Or (Supplementary Table 8). Among the InOr–Or differences, several pathways associated with the degradation of aromatic compounds, such as protocatechuate, gallate, and vanillin, showed higher predicted relative abundance in Or. Together with the observed differences in TOC and bacterial taxa associated with organic-substrate utilization, these predicted profiles suggest differences in microbial carbon-use potential between the two farms. However, the specific substrates and microbial activities underlying these patterns were not directly measured. Given the small sample size and the predictive nature of PICRUSt2, these findings should therefore be considered hypothesis-generating and warrant validation using shotgun metagenomic or metatranscriptomic approaches.

In addition, exploratory metabolomic profiling of pooled coffee bean samples indicated relatively higher abundances of several defense-related secondary metabolites in the InOr sample, including alkaloids, phenolic acids, coumarins, and the cyanogenic glycoside lotaustralin (Supplementary Figure 6; Supplementary Table 11). These compounds are commonly associated with plant defense and stress adaptation (Vaddadi and Parvatam, 2015) and may reflect differences in plant physiological responses to environmental conditions among the sampled farms (Yang et al., 2018). However, because metabolomic profiling was performed using pooled biological material from each farm, these observed differences should be regarded as exploratory observations rather than statistically validated farm-level differences. Independent biological replication across multiple farms will be necessary to confirm these metabolomic patterns and their relationships with microbiome variation.

Taken together, our findings show that the three farms with contrasting management histories, including conventional and organic farming, differed in rhizosphere bacterial community structure and predicted functional potential, with the greatest differences observed between the conventional and organic farms, which also differed in measured soil chemical properties. However, because each management category was represented by only one farm, the observed microbiome differences cannot be disentangled from farm-specific environmental and historical factors, such as soil properties, local microclimate, topography, and management history. Furthermore, because all samples were collected at a single time point during the harvest season, the observed microbial communities represent a temporal snapshot rather than long-term community dynamics. Consequently, the temporal stability of the observed patterns remains uncertain and should be interpreted with caution. Therefore, these observations should not be interpreted as causal or general effects of farming practices, but rather as differences observed among the three sampled farms with contrasting management histories. Nevertheless, field-based observational studies can provide valuable insights into complex agricultural systems under real production conditions, where environmental and management variability is difficult to reproduce in controlled experiments (Fujiwara et al., 2025). Thus, this study can complement controlled experiments by capturing real-world patterns and generating new testable hypotheses. In this context, when site and treatment are confounded, restricting the scope of inference and using observed patterns to formulate hypotheses for subsequent replicated studies has been advocated in ecological research (Davies and Gray, 2015). Future studies involving multiple independent farms per management category will therefore be needed to disentangle management effects from site-specific variation and evaluate the reproducibility and broader applicability of the microbial patterns identified here.

### The overlap between rhizosphere and fruit-associated bacterial communities in *Coffea canephora*

Comparison of alpha diversity between the rhizosphere and fruit-associated bacterial communities revealed that the rhizosphere harbored a more taxonomically diverse community, enriched in both rare and common bacterial taxa, whereas the fruit exhibited lower richness but greater evenness in taxa distribution (Figure 2, Supplementary Figures 1, 2). The present results are consistent with previous findings, where the rhizosphere of *C. arabica* (Veloso et al., 2020; Andrade et al., 2023) and *C. canephora* (Gomes et al., 2024) harbors a more diverse and abundant microbial community compared to the phyllosphere or fruit surface. This trend appears to be robust regardless of variations in soil nutrient composition or geographical conditions (Veloso et al., 2020; Andrade et al., 2023; Dos Santos Gomes et al., 2024) and plant species (Zhou et al., 2019), suggesting that rhizosphere bacteria may play important roles in nutrient cycling, plant health, and stress tolerance (Berg and Smalla, 2009; Chaparro et al., 2014). By contrast, the fruit surface represents a more specialized niche enriched in copiotrophic and fast-growing taxa able to exploit simple sugars (De Bruyn et al., 2017). Taken together, the rhizosphere represents a diverse microbial reservoir associated with plant–microbe interactions in *Coffea canephora*.

To further dissect the relationship between belowground and aboveground microbiomes, we examined their taxonomic overlap (Figure 4a). Our analysis revealed that approximately one third of the taxa detected across the rhizosphere and fruit were shared between the two compartments, indicating a substantial overlap between belowground and aboveground microbiomes in *C. canephora*. Shared genera included *Bacillus*, *Burkholderia*, *Citrobacter*, *Enterobacter*, *Gluconobacter*, *Kocuria*, *Lactobacillus*, *Leuconostoc*, *Kozakia*, *Pseudomonas*, *Serratia*, *Stenotrophomonas*, and *Streptomyces*, encompassing both root- and fruit-associated compartments. Notably, *Bacillus, Citrobacter, Enterobacter, Pseudomonas, Serratia,* and *Stenotrophomonas* have been consistently reported across coffee-growing regions worldwide (Duong et al., 2020), underscoring their ecological importance in coffee agroecosystems. Moreover, lactic acid bacteria (LAB) and acetic acid bacteria (AAB), which are central to coffee fermentation and strongly influence the sensory profile of the final beverage (Elhalis et al., 2023; Todhanakasem et al., 2024), were also detected in both rhizosphere and fruit compartments, suggesting potential ecological links between the two compartments. This is consistent with Costa et al. (2024), which demonstrated that a portion of fruit-associated microbial communities in *C. arabica* originate from the root zone and subsequently influence postharvest processing and quality outcomes. Comparative evidence from studies in other plant species supports these findings. In grapevine (*Vitis vinifera*), roots and leaves harbor overlapping microbial taxa under field conditions, suggesting that certain lineages colonize both belowground and aerial tissues (Zarraonaindia et al., 2015). Similarly, studies in *Arabidopsis thaliana* and bamboo have shown that root and leaf microbiota not only share taxa but also display convergence in genome-encoded functional traits and metabolic repertoires (Bai et al., 2015; Zheng and Lin, 2020).

The origin of aboveground plant microbiota remains a subject of ongoing debate. In the present study, rhizosphere-shared genera dominated the fruit-associated microbiome across all farms (>85% relative abundance), reaching the highest proportion in InOr (93.6%), followed by TranOr (89.2%) and Or (87.8%) (Figure 4b), indicating substantial taxonomic correspondence between rhizosphere and fruit-associated microbiomes. At the same time, previous studies have proposed that bulk soil acts as a primary microbial reservoir for aboveground plant microbiota (Zhou et al., 2021; Tkacz et al., 2020). Therefore, the overlap observed between rhizosphere and fruit microbiota may reflect shared recruitment from surrounding soil environments rather than direct microbial transmission. For coffee plants, particularly Coffea canephora, the ecological relationships and potential transmission dynamics among bulk soil, rhizosphere, and fruit-associated microbiomes remain poorly understood. Further investigations using controlled greenhouse experiments and larger-scale field studies will be important to clarify the origins and transmission pathways of microbiota across different coffee plant compartments.

## Conclusion

This study compared rhizosphere and fruit-associated microbiomes across three *Coffea canephora* farms representing conventional, transitional organic, and organic management histories. Distinct bacterial community compositions and predicted functional potentials were observed among the examined farms, with larger microbiome differences detected between the transitional organic and organic farms than between the conventional and transitional organic farms. In addition, substantial taxonomic overlap was identified between rhizosphere and fruit-associated microbiomes, suggesting potential ecological links between belowground and aboveground microbial communities. Although the present study was based on a limited number of farms and does not allow separation of management effects from farm-specific factors, these findings provide exploratory insights into microbiome variation across coffee production systems and highlight the potential importance of plant–soil–microbe interactions in sustainable coffee cultivation. The microbial patterns identified in this study should be regarded as candidate patterns for validation in future independently replicated studies.

## Data Availability

The datasets presented in this study can be found in online repositories. The names of the repository/repositories and accession number(s) can be found in the article/Supplementary material.

## Footnotes

1
www.metaboanalyst.ca

## Supplementary material

SUPPLEMENTARY FIGURE 1 Alpha diversity of rhizosphere and fruit microbiomes under different farms. **(a–c)** Chao1 index, observed features, and Pielou’s evenness of rhizosphere bacterial communities; **(d–f)** Chao1 index, observed features, and Pielou’s evenness of fruit-associated bacterial communities in Conventional, Transitional organic, and Organic farms. Sample size per group: *n* = 3. Statistical comparisons were conducted using the Tukey-HSD test, and no significant differences were observed among farmings.

SUPPLEMENTARY FIGURE 2 Alpha diversity of microbiomes in rhizosphere and fruit environments. **(a)** Chao1 index, **(b)** Observed features, **(c)** Pielou’s evenness, and **(d)** Shannon entropy. Sample size per compartment: *n* = 9 (three samples from each of the three farms). Statistical comparisons of alpha diversity were conducted using Tukey’s HSD test, revealing significant differences (*p* < 0.05) among rhizosphere and fruit microbiomes for all indices except Pielou’s evenness (*p* = 0.0576).

SUPPLEMENTARY FIGURE 3 Relative abundance of the top 20 bacterial genera in rhizosphere and fruit microbiomes across three farms. Stacked bar plots illustrate the relative abundance (%) of the most abundant bacterial genera identified in each sample. Each bar represents an individual sample, labeled according to farm and sample type (R corresponds to a rhizosphere sample and F corresponds to a fruit sample). Distinct colors represent different bacterial genera.

SUPPLEMENTARY FIGURE 4 The PLS-DA score plot illustrates the separation of samples based on their metabolite profiles. Sample size per group was *n* = 3. The axes represent the first two latent variables (PLS1 and PLS2), which explain the largest proportion of variance related to group discrimination. Ellipses indicate the 95% confidence interval for each group.

SUPPLEMENTARY FIGURE 5 Comparative heatmap of coffee bean metabolite profiles in each farm. Columns represent individual samples, while rows correspond to identified metabolite compounds. The color gradient reflects relative compound abundance, ranging from high (red) to low (blue). The dendrogram above the heatmap illustrates sample clustering based on metabolite profile similarity. The dendrogram on the left side of the heatmap shows clustering of metabolite compounds based on their abundance patterns. Sample size per group: *n* = 3.

SUPPLEMENTARY FIGURE 6 VIP score plot derived from PLS-DA analysis of coffee bean metabolites across conventional, transitional organic, and organic farms. Rows correspond to individual metabolite compounds, ranked by their Variable Importance in Projection (VIP) scores derived from the PLS-DA model. The scatter plot displays each metabolite’s VIP score, with higher scores indicating greater importance in differentiating between the three groups. The adjacent color bars indicate the relative abundance of each metabolite, with red representing high abundance and blue representing low abundance.

SUPPLEMENTARY TABLE 1 Sample metadata used for 16S rRNA gene amplicon analysis.

SUPPLEMENTARY TABLE 2 Soil chemical parameters, units, and analytical methods used in this study.

SUPPLEMENTARY TABLE 3 Soil pH values recorded from three beneath-canopy sampling sites of individual trees in each farm (*n* = 9 trees per farm).

SUPPLEMENTARY TABLE 4 Sequencing quality metrics for all samples.

SUPPLEMENTARY TABLE 5 Sample-level taxonomic relative abundances and alpha-diversity indices of rhizosphere bacterial communities. Taxonomic relative abundances are provided in separate sheets at the phylum, class, order, family, genus, and species levels. Alpha-diversity indices for each individual sample are provided in a separate sheet.

SUPPLEMENTARY TABLE 6 Sample-level taxonomic relative abundances and alpha-diversity indices of fruit bacterial communities. Taxonomic relative abundances are provided in separate sheets at the phylum, class, order, family, genus, and species levels. Alpha-diversity indices for each individual sample are provided in a separate sheet.

SUPPLEMENTARY TABLE 7 ALDEx2 differential-abundance results for rhizosphere bacterial communities among farms. Pairwise comparisons are provided for InOr vs. TranOr, InOr vs. Or, and TranOr vs. Or at the species-level feature, genus, and family levels in separate sheets. For each taxonomic feature, the table reports relative abundance, log-ratio abundance, between-group difference, effect size, 95% confidence interval of the effect size, and whether the feature met the differential-abundance criteria. Positive difference values indicate higher abundance in the first farm listed, whereas negative values indicate higher abundance in the second farm listed. Features were considered differentially abundant when the absolute effect size was > 1, the absolute between-group difference was > 1, and the effect-size confidence interval did not cross zero.

SUPPLEMENTARY TABLE 8 ALDEx2 differential-abundance results for PICRUSt2-predicted functional pathways in rhizosphere microbiomes among farms. Pairwise comparisons are provided for InOr vs. TranOr, InOr vs. Or, and TranOr vs. Or in separate sheets. For each predicted pathway, the table reports relative abundance, log-ratio abundance, between-group difference, effect size, 95% confidence interval of the effect size, and whether the pathway met the differential-abundance criteria. Positive difference values indicate higher predicted relative abundance in the first farm listed, whereas negative values indicate higher predicted relative abundance in the second farm listed. Pathways were considered differentially abundant when the absolute effect size was > 1, the absolute between-group difference was > 1, and the effect-size confidence interval did not cross zero.

SUPPLEMENTARY TABLE 9 ALDEx2 differential-abundance results for fruit-associated bacterial communities among farms. Pairwise comparisons are provided for InOr vs. TranOr, InOr vs. Or, and TranOr vs. Or at the species-level feature, genus, and family levels in separate sheets. For each taxonomic feature, the table reports relative abundance, log-ratio abundance, between-group difference, effect size, 95% confidence interval of the effect size, and whether the feature met the differential-abundance criteria. Positive difference values indicate higher abundance in the first farm listed, whereas negative values indicate higher abundance in the second farm listed. Features were considered differentially abundant when the absolute effect size was > 1, the absolute between-group difference was > 1, and the effect-size confidence interval did not cross zero.

SUPPLEMENTARY TABLE 10 Summary of compound annotation results and corresponding analytical parameters across samples.

SUPPLEMENTARY TABLE 11 VIP scores of metabolites contributing to class discrimination in PLS-DA model.

SUPPLEMENTARY TABLE 12 Occurrence patterns of microbial taxa across rhizosphere and fruit microbiomes within each farm. Separate sheets are provided for InOr, TranOr, and Or at the genus and species-level resolutions. Taxa were classified as “shared” when detected in both rhizosphere and fruit microbiomes, “fruit only” when detected exclusively in fruit samples, or “rhizosphere only” when detected exclusively in rhizosphere samples. A taxon was considered present in a compartment when detected in at least one of the three replicate samples. Mean relative abundance in the fruit microbiome (%) was calculated from the three fruit samples within each farm.
